# Efficacy of UFT combined with radiotherapy in advanced pancreatic cancer: a systematic review and meta-analysis

**DOI:** 10.3389/fonc.2026.1915790

**Published:** 2026-08-31

**Authors:** Xiaoyun Shang, Xiaobo Wang, Jiawei Cen, Fan Yang, Juntao Li, Hui Zhao

**Affiliations:** First Department of Hepatobiliary Surgery, The Fourth Affiliated Hospital of Dali University., Chuxiong, Yunnan, China

**Keywords:** advanced pancreatic cancer, adverse drug reaction, chemoradiotherapy, meta-analysis, objective response rate, tegafur–uracil

## Abstract

**Background:**

Tegafur–uracil (UFT) combined with radiotherapy is a treatment option for advanced pancreatic cancer. The purpose of this study was to evaluate the overall efficacy and safety of this combination therapy and explore potential factors associated with treatment outcomes.

**Method:**

The meta-analysis was in line with the PRISMA 2020 and MOOSE guidelines. Web of Science, PubMed, Scopus and Embase databases were searched for eligible studies. The outcomes variables were objective response rate (ORR) and adverse drug reaction (ADR). Pooled proportions were synthesized using meta-analysis method with a random-effect model. Influential publication was determined by performing a sensitivity analysis. In addition, the potential sources of heterogeneity were examined using subgroup analyses. Publication bias was assessed using a funnel plot, Begg’s and Egger’s tests.

**Results:**

The 7 observational studies, included for the SRMA, were conducted between 2004 and 2016, involving secondary data of 237 patients who underwent UFT combined with radiotherapy. We found that the pooled ORR of UFT combined with radiotherapy was 38% (95% CI: 13% to 67%, *P* < 0.001), with high heterogeneity (I^2^ = 94.25%, *P* < 0.001). The pooled ADR was 21% (95% CI: 10% to 34%, *P* < 0.001), with a high heterogeneity (I^2^ = 76.25%, *P* < 0.001). Subgroup analyses revealed that patient age and UFT dosage were critical factors influencing outcomes. Specifically, compared with other doses, UFT (250 mg/m2/day) had a higher ORR (69%, 95% CI: 56% to 81%) and a lower ADR (6%, 95% CI: 1% to 14%).

**Conclusion:**

UFT combined with radiotherapy is an effective and tolerable treatment strategy for patients with advanced pancreatic cancer, with a pooled ORR of 38% and an ADR of 21%. Age of the patients and drug dosage were significant determinants of treatment outcomes. However, further research is needed to confirm these findings in large-scale randomized controlled trials.

**Systematic Review Registration:**

https://www.crd.york.ac.uk/PROSPERO/view/CRD420261377332, identifier, CRD420261377332.

## 1 Introduction

Pancreatic cancer is a lethal malignancy with an increasing global incidence, currently ranking as the sixth leading cause of cancer mortality and accounting for nearly 5% of all cancer deaths (1). In the United States, approximately 67,000 newly diagnosed cases of pancreatic cancer were reported in 2024, reflecting an annual incidence increase rate of 1.1% (2). The median overall survival (OS) for patients with pancreatic cancer remains limited to merely a few months, and the 5-year OS rate is only 5%-13% (3). In addition, mortality rates have continued to increase since the 1930s, a trend partially driven by the obesity epidemic (4). The primary limitation in improving the survival rate of patients with pancreatic carcinoma lies in the challenges associated with early screening (5). The Cancer of the Pancreas Screening Study demonstrated that surveillance facilitates early detection, with nearly 80% of cancers diagnosed at stage I among monitored individuals, while among non-monitored individuals 85% of the cancers were diagnosed at stage IV (6).

Early-stage pancreatic cancer typically manifests with nonspecific symptoms, hindering early diagnosis. As a result, most patients present with advanced disease, precluding curative interventions (7). Surgical resection is the only treatment that is potentially curative. However, this approach remains unfeasible for over 80% of patients, owing to the advanced stage of disease at initial diagnosis (8). Pancreatic cancer without distant metastases is broadly stratified into resectable, borderline resectable, and locally advanced categories, on the basis of the tumor involvement with major vascular structures (9). For the majority of patients with metastatic disease, the 5-year OS rate remains below 10%. Since the majority of patients present with locally advanced or metastatic disease, systemic chemotherapy remains the clinical mainstay of treatment. Currently, two primary systemic chemotherapy regimens have demonstrated clinical efficacy, viz., multiagent 5-fluorouracil (5-FU)-based therapies and gemcitabine-based chemotherapy (10). The introduction of combination chemotherapy regimens, such as FOLFIRINOX, represents a significant advancement that has extended the median overall survival for patients with metastatic disease (11). Currently, emerging combination strategies are being actively investigated to overcome therapeutic resistance (12). However, its therapeutic benefit is modest and frequently offset by substantial toxicity (13). In contrast, the use of chemoradiotherapy is less invasive and is associated with lower rates of major complications. Concurrent chemoradiotherapy (CCRT) is the current standard of care for advanced pancreatic cancer, offering both survival benefits and effective local tumor control (14). Studies have confirmed that compared with upfront surgery, neoadjuvant chemoradiotherapy is associated with longer progression-free survival (PFS) and OS (15, 16).

Oral tegafur–uracil (UFT) consists of tegafur, a prodrug of 5-FU, paired with uracil to inhibit dihydropyrimidine dehydrogenase, thereby preventing the enzymatic degradation of 5-FU and enhancing its therapeutic efficacy. It has demonstrated significant clinical antitumor activity across various malignancies, particularly in the treatment of gastrointestinal cancers (17). The National Comprehensive Cancer Network (NCCN) guidelines recommend adjuvant 5-FU-based chemotherapy following chemoradiation therapy (18). The ESPAC trial was the first to demonstrate an overall survival benefit with adjuvant 5-FU compared with observation alone (19). Despite the potential of UFT, its efficacy when combined with radiotherapy lacks a systematic evaluation, leaving its feasibility and response determinants undefined. Hence, we conducted a meta-analysis to rigorously evaluate the objective response rate (ORR) and safety profile of UFT-based chemoradiation.

## 2 Methods

The study was in line with the Preferred Reporting Items for Systematic Reviews and Meta-Analysis (PRISMA) and MOOSE guidelines (20, 21). The protocol for this systematic review and meta-analysis was registered on the PROSPERO website (ID: CRD420261377332).

### 2.1 Eligibility criteria

Studies evaluating the efficacy and safety of UFT combined with radiotherapy in advanced pancreatic cancer were included. Eligible studies enrolled adults with confirmed advanced pancreatic cancer treated with UFT plus radiotherapy. Both comparator and single-arm studies were eligible. Comparator groups in the included studies included placebo, radiotherapy alone or other chemotherapy regimens. To be included, studies were required to report at least one of the following outcomes: ORR, OS, PFS, or safety. Studies meeting any of the following criteria were excluded: (1) laboratory research; (2) non-English language; and (3) unavailable full text. The publication date, study design, and sample size were not used as exclusion criteria.

### 2.2 Search strategy

In accordance with the PICO strategy for the review question, the keywords included “patients with advanced pancreatic cancer” (P), “UFT combined with radiotherapy” and “efficacy or safety”. Web of Science, PubMed, Scopus and Embase databases were screened from the inception of the databases to April 2026. References in reviews and meta-analyses on this topic were also searched. The search query was: (Pancreatic Neoplasms OR pancreatic cancer OR pancreatic tumor OR pancreatic carcinoma) AND (“UFT” OR Tegafur OR Uracil) AND (Radiotherapy OR Radiation therapy OR chemoradiotherapy).

### 2.3 Study selection

Databases were searched separately, and the retrieved records were then imported into EndNote software, where duplicate entries were identified and removed. Titles and abstracts were subsequently screened for relevance to the objectives of the study. Full-text articles were then assessed against the predefined inclusion and exclusion criteria. Two reviewers independently screened records, with disagreements resolved through discussion or adjudication by a third reviewer.

### 2.4 Quality assessment

Two reviewers independently evaluated the risk of bias in the included studies by using the Newcastle–Ottawa Scale (NOS) checklist. The NOS checklist comprises 7 items, each of which receives 1 point, except for the comparison item, which can receive a maximum of 2 points. Studies scoring below 5 were considered to have a high risk of bias (22).

### 2.5 Data extraction and synthesis

After the quality of the included studies was evaluated, two reviewers independently extracted the data, and a third reviewer verified the accuracy of the extracted information. Data were extracted using a standardized form covering author, year, country, study design, duration, sample size, mean age, tumor size, dose of UFT and radiotherapy protocol. The objective response rate (ORR) and adverse drug reactions (ADRs) with 95% confidence intervals (95% CIs) were computed with a random-effects model. In accordance with the Response Evaluation Criteria in Solid Tumors (RECIST), the ORR was defined as the proportion of patients whose best overall response was either complete response (CR) or partial response (PR). In addition, ADRs were specifically defined as adverse events ≥ grade 3. Heterogeneity was quantified by the I^2^ statistic and Cochran’s Q test. The values of 25%, 50%, and 75% corresponded to cutoff points for low, moderate, and high heterogeneity, respectively. Owing to the high degree of heterogeneity, a sensitivity analysis was performed to identify and eliminate the influential publications. In addition, potential sources of heterogeneity were identified by subgroup analysis. Publication bias was assessed by using a funnel plot and Begg’s and Egger’s tests.

### 2.6 Statistical analysis

The results were pooled with STATA-16 software by employing the “metaprop”, “metaninf”, “metabias” and “metafunnel” packages. For the single-arm meta-analysis of ORR and ADRs, we employed the Freeman–Tukey double arcsine transformation to stabilize variances across studies, particularly given the presence of low event rates in some included studies. All the tests were two-tailed, and a *P*-value < 0.05 was considered to be statistically significant.

## 3 Results

This study is a comprehensive meta-analysis to investigate overall efficacy and safety of UFT combined with radiotherapy in patients with advanced pancreatic cancer and explore the potential factors associated with the treatment outcomes.

### 3.1 Study selection

**Figure 1:**
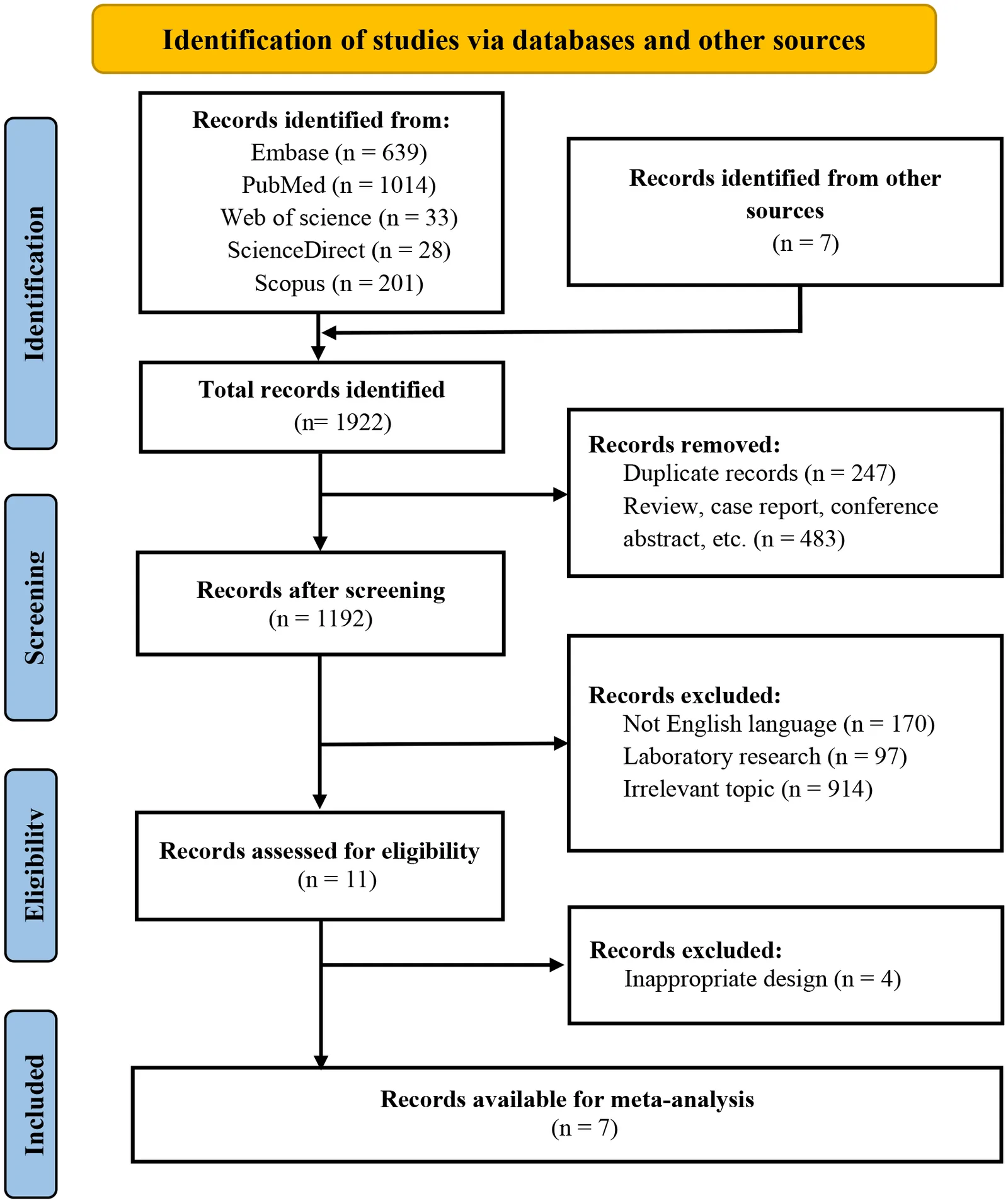
PRISMA flowchart of 7 included studies.

The process of selection is shown in **Figure 1**. An initial search yielded 1,915 records from electronic databases, supplemented by 7 additional studies identified through manual screening of reference lists. After removing 247 duplicates and 483 irrelevant records, 1,192 records were screened. After the exclusion of 170 non-English studies, 97 laboratory studies, and 914 studies with irrelevant topics, 11 full-text articles were assessed for eligibility. Ultimately, 7 studies were included in the final analysis (23–29).

### 3.2 Study characteristics and quality assessment

The 7 included observational studies were conducted between 2004 and 2016, covering a total of 237 patients who underwent UFT combined with radiotherapy. The sample size varied between 12 and 79 patients. The patients’ ages ranged from 58 to 64 years. Other detailed information about the study characteristics is presented in **Table 1**. In addition, the scores of the included studies were not less than 5 according to the NOS checklist, and none of them was excluded (**Supplementary Table 1**).

**Table 1:** Summarized characteristics of 7 included studies.

Study ID	Country	Design	Duration	Simple size	Mean age	Mean tumor size	Dose of UFT	Radiotherapy
Bjerregaard, J., et al. (2009) (23)	Denmark	Single arm	2001-2005	54	63 years	3.6 cm	300 mg/m^2^/day	50 Gy/27 fractions
Calvo, F, et al. (2004) (24)	Spain	Single arm	1998-2001	15	61 years	> 6 cm	300 mg/m^2^/day	45-50Gy/27 fractions
Childs, I. H., et al. (2000) (25)	USA	Single arm	1997-1998	12	58 years	> 2 cm	150 mg/m^2^/day	45 Gy/25 fractions
Khan, K., et al. (2016) (26)	UK	Cohort	2014-2015	17	61 years	> 2 cm	250 mg/m^2^/day	45Gy/25 fractions
Morak, M., et al. (2011) (27)	UK	Single arm	2003-2007	79	62 years	4 cm	300 mg/m^2^/day	50 Gy/20 fractions
Nio, Y., et al. (2005) (28)	Japan	Cohort	2001-2004	22	64 years	> 4 cm	300 mg/m^2^/day	40–60 Gy/25 fractions
Zhang, L., et al. (2008) (29)	China	Cohort	2001-2006	38	61 years	> 2 cm	250 mg/m^2^/day	25–50 Gy/10 fractions

### 3.3 Overall efficacy and related factors of UFT combined with radiotherapy in advanced pancreatic cancer

**Figure 2:**
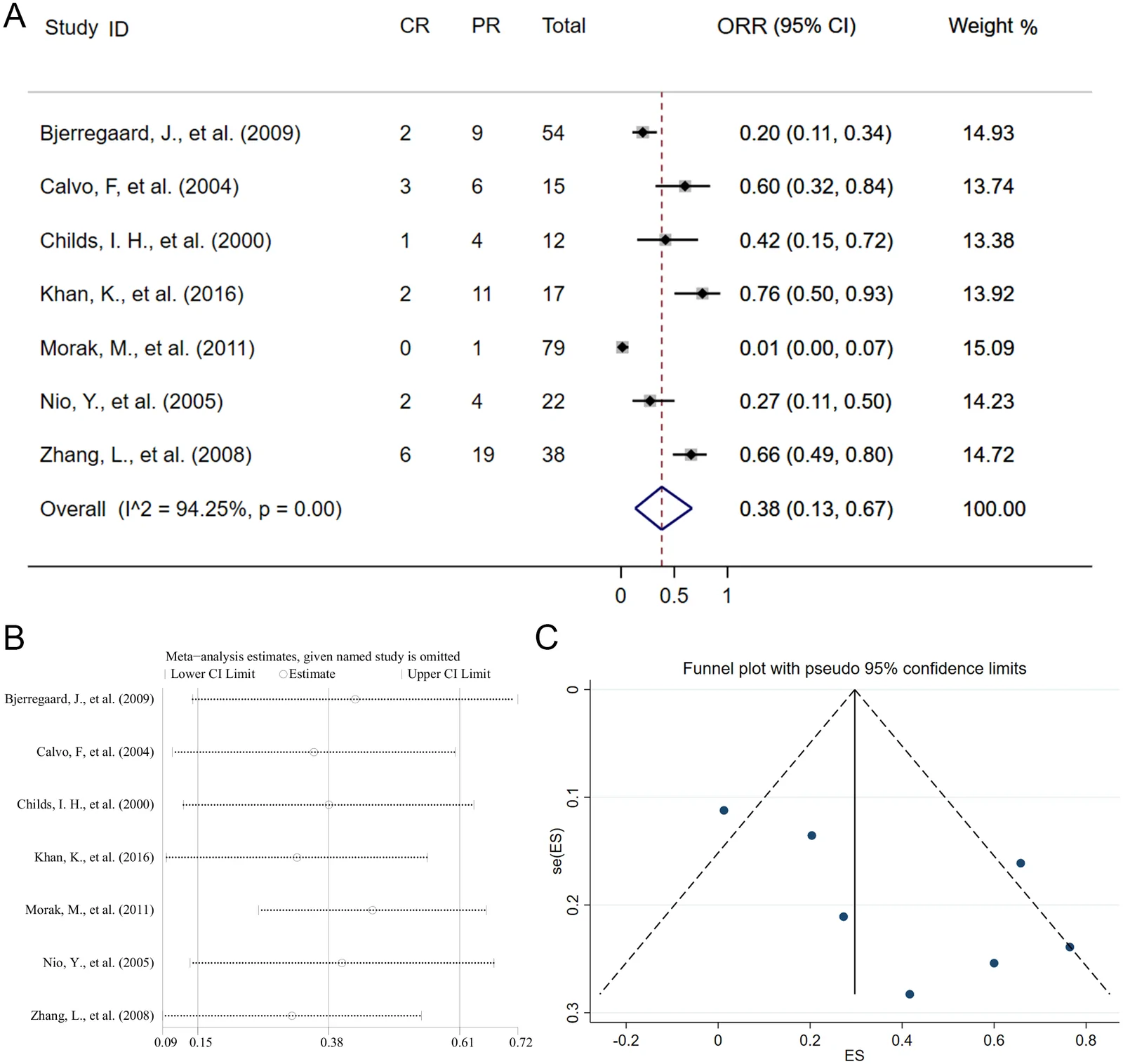
Overall efficiency and relative factors of UFT combined with radiotherapy in advanced pancreatic cancer. **(A)** Forest plot of the pooled ORR; **(B)** Sensitivity analysis for the pooled ORR; **(C)** Funnel plot for assessing publication bias.

The pooled ORR of UFT combined with radiotherapy was 38% (95% CI: 13% to 67%, *P* < 0.001). Among the included studies, the lowest ORR (1%) was reported by Morak, M., et al. (2011), whereas the highest ORR (66%) was reported by Zhang, L., et al. (2008). Heterogeneity among these studies was high (I^2^ = 94.25%, *P* < 0.001) (**Figure 2A**). However, the sensitivity analysis generated stable summary estimates without detecting influential publications (**Figure 2B**). The results of the subgroup analysis revealed that the possible sources of heterogeneity were the patient’s mean age and dose of UFT. The cutoffs of 61 years for age and 22 for sample size were defined on the basis of the median values of the total pooled population and the included studies, respectively. Patients with a mean age ≤61 years (ORR = 63%, 95% CI: 51% to 75%, interaction *P* value = 0.007) had a higher ORR than those with a mean age >61 years did (ORR = 13%, 95% CI: 0% to 37%, interaction *P* value < 0.001). The patients with a dosage of UFT = 250 mg/m^2^/day had the highest ORR (ORR = 69%, 95% CI: 56% to 81%, interaction *P* value = 0.007). However, the study design (interaction *P* value = 0.141) and sample size (interaction *P* value = 0.267) were not possible sources of heterogeneity (**Table 2**). Furthermore, the results of the funnel plot (**Figure 2C**), Begg’s (z = 0.90; *P* value = 0.368) and Egger’s (t = 1.85; *P* value = 0.064) tests did not reveal statistically significant publication bias. However, given the limited number of included studies, these results should be interpreted with caution because of low statistical power.

**Table 2:** Subgroup analyses for sources of heterogeneity.

Outcome	Potential factors	Subgroup	Effect (95% CI)	Interaction *p*-value
ORR	Mean age	≤ 61 years	0.63 (0.51, 0.75)	**<0.001**
		> 61 years	0.13 (0.00, 0.37)	
	UFT dosage	150 mg/m^2^/day	0.42 (0.15, 0.72)	**0.007**
		250 mg/m^2^/day	0.69 (0.56, 0.81)	
		300 mg/m^2^/day	0.22 (0.03, 0.50)	
	Study design	Single arm	0.25 (0.03, 0.57)	0.141
		Cohort	0.57 (0.29, 0.83)	
	Sample size	≤22	0.51 (0.28, 0.74)	0.267
		>22	0.24 (0.00, 0.67)	
ADR	Mean age	≤ 61 years	0.17 (0.02, 0.41)	0.621
		> 61 years	0.25 (0.12, 0.41)	
	UFT dosage	150 mg/m^2^/day	0.17 (0.02, 0.48)	**0.018**
		250 mg/m^2^/day	0.06 (0.01, 0.14)	
		300 mg/m^2^/day	0.29 (0.15, 0.45)	
	Study design	Single arm	0.26 (0.12, 0.42)	0.405
		Cohort	0.15 (0.01, 0.38)	
	Sample size	≤22	0.28 (0.16, 0.42)	0.203
		>22	0.15 (0.02, 0.34)	

ADR, adverse drug reaction; ORR, objective response rate; UFT, tegafur–uracil.

Bold values indicate statistical significance.

### 3.4 Safety and relative factors of UFT combined with radiotherapy in advanced pancreatic cancer

**Figure 3:**
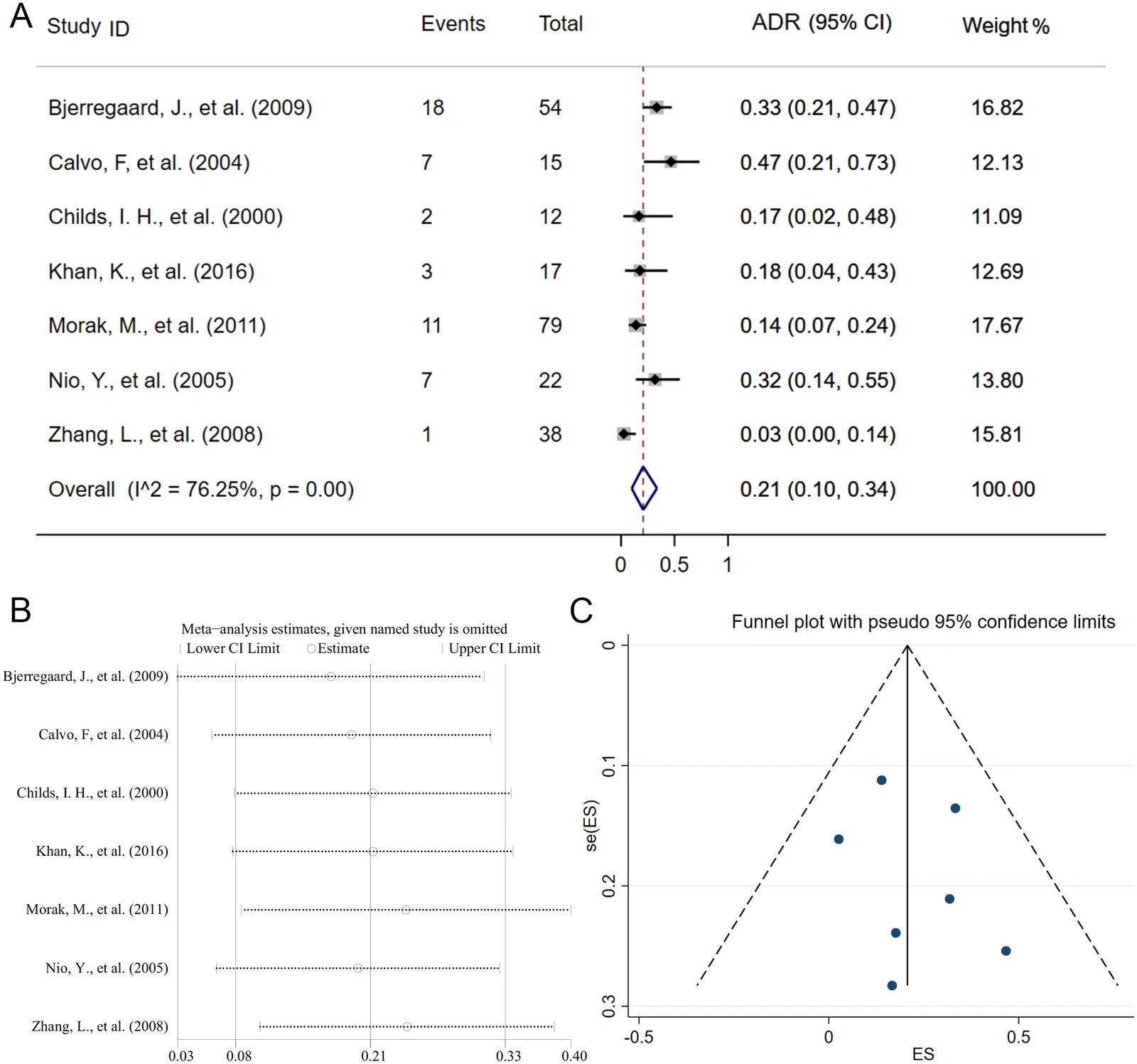
Safety and relative factors of UFT combined with radiotherapy in advanced pancreatic cancer. **(A)** Forest plot of the pooled ADR; **(B)** Sensitivity analysis for the pooled ADR; **(C)** Funnel plot for assessing publication bias.

The pooled ADR of UFT combined with radiotherapy was 21% (95% CI: 10% to 34%, *P* < 0.001). Among the included studies, the lowest ADR was reported by Zhang, L., et al. (2008) at 3%, while the highest ADR was observed in Calvo, F, et al. (2004) at 47%. Heterogeneity among these studies was high (I^2^ = 76.25%, *P* < 0.001) (**Figure 3A**). However, the sensitivity analysis generated stable summary estimates without detecting influential publications (**Figure 3B**). The results of the subgroup analysis revealed that the possible source of heterogeneity was the dose of UFT. The patients for whom the dosage of UFT was 250 mg/m^2^/day had the lowest ADR (ADR = 6%, 95% CI: 1% to 14%, interaction *P* value = 0.018). However, the patient’s mean age (interaction *P* value = 0.621), study design (interaction *P* value = 0.405) and sample size (interaction *P* value = 0.203) were not possible sources of heterogeneity (**Table 2**). Although the statistical power is limited by the small number of studies, Begg’s (z = 0.00, *P* value = 1.000) and Egger’s (t = 0.57, *P* value = 0.566) tests did not suggest significant publication bias, which was further supported by the visual symmetry of the funnel plot (**Figure 3C**).

## 4 Discussion

Pancreatic cancer remains a lethal malignancy with a poor prognosis and is currently the sixth leading cause of cancer mortality, with a 5-year overall survival rate of merely 5%-13% (1, 3). The primary challenge lies in early detection; as symptoms are often nonspecific and, the majority of patients present with advanced disease precluding curative resection (5, 7, 8). While systemic chemotherapy regimens such as FOLFIRINOX have improved outcomes, their benefits are frequently offset by substantial toxicity (11–13). Consequently, CCRT has emerged as a standard of care for advanced cases. It offers a less invasive alternative with lower complication rates while ensuring effective local tumor control (14). Furthermore, compared with upfront surgery, neoadjuvant chemoradiotherapy has demonstrated superior PFS and OS, highlighting its critical role in managing advanced disease (15, 16).

This meta-analysis demonstrated that UFT combined with radiotherapy yields a pooled ORR of 38% and a pooled ADR of 21% in advanced pancreatic cancer patients. Subgroup analyses revealed that patient age and UFT dosage were critical factors influencing outcomes. Specifically, patients with a mean age of ≤61 years had a significantly greater ORR (63%) than older patients did. Age is the single most significant nongenetic demographic risk factor for pancreatic cancer, with 80% of cases occurring predominantly in individuals aged 60 to 80 years (30). Furthermore, compared with other dosages, a UFT dosage of 250 mg/m²/day demonstrated a favorable balance between efficacy (ORR = 69%) and toxicity (ADR = 6%). However, only two studies contributed to the 250 mg/m²/day subgroup. Thus, the finding should be considered exploratory and hypothesis-generating. These findings suggest that UFT-based chemoradiotherapy offers a favorable therapeutic window, particularly in younger patients or when it is administered at optimized dosages.

UFT is a prescription medication that was first introduced in Japan during the 1980s. Tegafur is primarily converted to 5-FU by hepatic cytochrome P450 enzymes, whereas uracil inhibits the activity of dihydropyrimidine dehydrogenase, which serves as the rate-limiting enzyme in the degradation of 5-FU (31). Preclinical studies indicate that the efficacy of radiotherapy can be either potentiated or compromised by specific chemotherapy agents, contingent upon dosing, administration schedules, and their distinct mechanisms of action (32). For example, a standard UFT regimen for advanced colorectal cancer consists of tegafur at a recommended dosage of 300 mg/m² daily, combined with leucovorin (75 mg/day), administered for 28 days followed by a 7-day rest period before subsequent cycles (33). While oral UFT provides a convenient alternative to prolonged 5-FU infusions, it is associated with considerable inter- and intra-individual variability in plasma drug concentrations (34). Circadian fluctuations in plasma 5-FU levels during prolonged infusion can lead to therapeutic failure, likely driven by the circadian rhythmicity of dihydropyrimidine dehydrogenase activity (35).

Several limitations of this study warrant consideration. First, the observational studies introduce potential selection bias and confounding factors (patient selection, radiotherapy protocols, etc.), which may affect the validity of our pooled estimates. Second, the limited number of included studies reduces statistical power, precluding reliable assessment of publication bias or comprehensive meta-regression for heterogeneity. Third, as all included studies were published between 2000 and 2016, the generalizability of their findings on the efficacy and safety of UFT combined with radiotherapy to contemporary clinical practice should be interpreted with caution. Finally, the included studies were predominantly single-arm studies without a control group, which imposes constraints on causal inference. Future randomized controlled trials are needed to validate these findings.

## 5 Conclusion

This meta-analysis revealed that UFT combined with radiotherapy has promising antitumor activity and a manageable safety profile in patients with advanced pancreatic cancer, with a pooled ORR of 38% and an ADR of 21%. Patient age and drug dosage are significant determinants of treatment outcomes. Specifically, a UFT dosage of 250 mg/m²/day is associated with favorable efficacy, maximizing tumor response while minimizing toxicity. Consequently, UFT combined with radiotherapy may represent a potential therapeutic option, although further validation in large-scale randomized controlled trials is warranted.

## Data Availability

The original contributions presented in the study are included in the article/**Supplementary Material**. Further inquiries can be directed to the corresponding author.
