# Supplementary material for: Efficacy of UFT combined with radiotherapy in advanced pancreatic cancer: a systematic review and meta-analysis
Source: Front Oncol. 2026 Aug 31;16:1915790. doi: 10.3389/fonc.2026.1915790 (PMC13572220; doi:10.3389/fonc.2026.1915790)
Supplement: Supplementary file 1 [file Table1.doc]

| **Study** | **Selection** | | | |  | **Comparability** |  | **Outcome** | | **Total score** |
| --- | --- | --- | --- | --- | --- | --- | --- | --- | --- | --- |
|  | Representativeness of the sample | Sample | Non-respondent | Ascertainment of the exposure |  | Confounding factors are controlled |  | Assessment of outcomes | Statistical test |  |
| Bjerregaard, J., et al. (2009) | * | * | * | * |  | - |  | * | * | 6 |
| Calvo, F, et al. (2004) | * | * | * | * |  | - |  | * | * | 6 |
| Childs, I. H., et al. (2000) | * | * | * | * |  | - |  | * | * | 6 |
| Khan, K., et al. (2016) | * | * | * | * |  | * |  | * | * | 7 |
| Morak, M., et al. (2011) | * | * | * | * |  | - |  | * | * | 6 |
| Nio, Y., et al. (2005) | * | * | * | * |  | * |  | * | * | 7 |
| Zhang, L., et al. (2008) | * | * | * | * |  | ** |  | * | * | 8 |

Table S1. Quality assessment based on the Newcastle-Ottawa Scale (NOS) checklist.
